# From prevention to progression: can belzutifan-based strategies deliver across the RCC disease continuum?

**DOI:** 10.1093/oncolo/oyag193

**Published:** 2026-05-16

**Authors:** Kathryn Beckermann

**Affiliations:** Medical Director of GU Clinical Research, Greco-Hainsworth Center for Research, Tennessee Oncology, Nashville, TN 37203, United States

**Keywords:** renal cell carcinoma, GU ASCO, belzutifan, HIF-2α, adjuvant therapy, LITESPARK-011, LITESPARK-022

## Abstract

HIF-2α signaling is a central driver of clear cell renal cell carcinoma biology. With regulatory approval of the HIF-2α inhibitor, belzutifan, in the third- and fourth-line refractory setting, therapeutic targeting of this pathway has entered clinical practice. At the 2026 ASCO GU Cancer Symposium, two randomized phase III trials evaluated earlier integration of belzutifan: LITESPARK-022 (belzutifan plus pembrolizumab vs pembrolizumab alone in the adjuvant setting) and LITESPARK-011 (belzutifan plus lenvatinib vs cabozantinib in the post-PD-(L)1 setting). LITESPARK-022 demonstrated a disease-free survival benefit (HR 0.72; 95% CI, 0.59-0.87; *P* = .0003), with early and sustained curve separation, though grade ≥3 adverse events doubled and overall survival data remain immature. LITESPARK-011 enrolled patients after progression on prior PD-(L)1 therapy in the metastatic setting. The combination improved median progression-free survival (PFS; 14.8 vs 10.7 months; HR 0.70), objective response rate (ORR; 52.6% vs 40%), and duration of response (23 vs 12 months), with a manageable toxicity profile. Together, these studies suggest that earlier targeting of HIF-2α biology may improve disease control and durability. However, patient selection, toxicity considerations, and the absence of mature OS data remain critical factors in determining the optimal role of belzutifan-based strategies across disease states.

## Graphical abstract

**Graphical Abstract:**
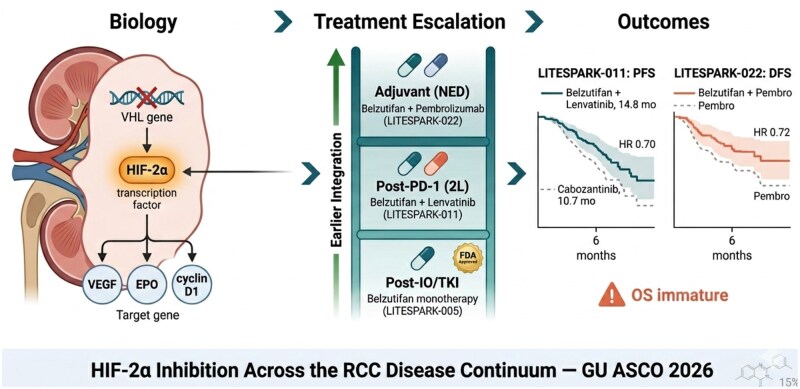

## Introduction

The biologic rationale for HIF-2α inhibition in RCC is well established, grounded in Nobel Prize-winning work defining the role of hypoxia sensing in tumorigenesis.^1^ HIF-2α is a principal downstream effector of VHL loss and a central driver of ccRCC pathophysiology. LITESPARK-005 established regulatory approval for belzutifan in the third- and fourth-line setting.^2^ The defining feature of that trial was the durability of benefit among responders, but the challenge of monotherapy in a heavily pretreated population was reflected in a 34% rate of primary progressive disease as best response. LITESPARK-011 and LITESPARK-022 tested whether earlier integration of belzutifan with established agents could improve efficacy and outcomes for patients.

## LITESPARK-022: adjuvant intensification in high-risk ccRCC

LITESPARK-022 randomized 1841 patients with resected high-risk ccRCC to receive pembrolizumab with or without belzutifan for one year.^3^ The study met its primary endpoint, demonstrating improved DFS (HR 0.72; 95% CI, 0.59-0.87; *P* = .0003), corresponding to an absolute ∼7% improvement at two years (Table 1). The DFS curves separated early and continued to diverge after completion of therapy, suggesting potential biologic activity beyond on-treatment suppression. However, no clear biomarker-defined subgroup with preferential benefit was identified.

**Table 1:** Efficacy and safety summary: LITESPARK-011 and LITESPARK-022, GU ASCO 2026.

	LITESPARK-011 Post-PD-1 Metastatic RCC • *N* = 747		LITESPARK-022 Adjuvant ccRCC • *N* = 1841	
	Belzutifan 120 mg + Lenvatinib 20 mg QD	Cabozantinib 60 mg QD	Belzutifan 120 mg + Pembrolizumab Q3W ×1 yr	Pembrolizumab Q3W ×1 yr
**Efficacy**				
** Primary endpoint HR (95% CI; *P*-value)**	**HR 0.70** (0.59-0.84) ***P* < .0001**	1.00 (comparator)	**HR 0.72** (0.59-0.87) ***P* = .0003**	1.00 (comparator)
** Median PFS/DFS (months)**	**14.8 mo**	10.7 mo	NR; 24-mo DFS 80.7%	NR; 24-mo DFS 73.7%
** Absolute risk reduction (at 2 years)**	**+4.1 mo median PFS**	—	**∼7% at 2 years**	—
** ORR**	**52.6%**	40.2%	N/A—adjuvant setting	N/A—adjuvant setting
** Primary progressive disease**	**5.7%**	5.9%	N/A	N/A
** Duration of response (median, months)**	**23.0 mo**	12.3 mo	N/A—adjuvant setting	N/A—adjuvant setting
** Overall survival**	**HR 0.85 (0.68-1.05)** *P* = .061 Median: 34.9 vs 27.6 mo—immature	27.6 mo (comparator)	**HR 0.78 (0.51-1.19)** *P* = .122 Median NR both arms—immature	NR (comparator)
** OS status**	**⚠ Did not cross α boundary**	—	**⚠ Did not cross α boundary**	—
**Safety & tolerability**				
** Grade ≥3 AEs (any)**	All-grade & Gr ≥3 comparable between arms	Comparable	**42.2% (belzutifan arm)**	17.9%
** Anemia, any grade (on-target, EPO↓)**	69%	25.6%	84% any grade 7% received ESA	11.4%
** Hypoxia (on-target)**	15.4% any grade Manageable; class effect	0%	4.6% grade 3 (limits ADLs) 3.3% received oxygen	Not a class effect
** Cardiac dysfunction (lenvatinib-related)**	7%	1.1%	N/A	N/A
** Dose reduction**	Belzutifan: 33% Lenvatinib: 66%	Cabozantinib: 77%	Belzutifan: 17%, anemia 3.5% hypoxia	Lower rate
** Treatment discontinuation**	Belzutifan: 17% Lenvatinib: 21% Combo overall: 11%	Cabozantinib: 11%	Belzutifan: 4% anemia 1.6% hypoxia	Low rate

Abbreviations: ADL, activities of daily living; AE, adverse event; ccRCC, clear cell renal cell carcinoma; DFS, disease-free survival; DOR, duration of response; EPO, erythropoietin; ESA, erythropoieitin stimulating agent; IMDC, International Metastatic RCC Database Consortium; NED, no evidence of disease; NR, not reached; ORR, objective response rate; OS, overall survival; PD, progressive disease; PFS, progression-free survival; QD, once daily; Q3W, every 3 weeks; Rx, treatment. **Green bold** = statistically significant or clinically favorable result. **Coral shading** = caution signal (OS immaturity; excess toxicity; absent QoL data). LITESPARK-011 comparator is cabozantinib 60 mg QD (CONTACT-03: ORR 41%, mPFS 10.8 mo, DOR 14.8 mo). LITESPARK-022 comparator is pembrolizumab alone (KEYNOTE-564 OS HR 0.66, 5-yr OS data). OS boundaries not crossed in either trial at GU ASCO 2026 presentation; manuscripts pending.

The clinical relevance of DFS improvement in this setting must be considered in context. Adjuvant pembrolizumab has already demonstrated an OS benefit, establishing a high bar for intensification strategies.^4^ In LITESPARK-022, OS remains immature and has not crossed the pre-specified boundary (HR 0.78; *P* = .12) (Table 1). In a population rendered disease-free by surgery, the central question is not only whether recurrence is delayed, but whether lethal recurrence is prevented.

Toxicity further complicates the risk-benefit calculus. Grade ≥3 adverse events increased substantially with the addition of belzutifan (42.2% vs 17.9%). On-target toxicities, including anemia requiring dose interruptions (25%) and reductions (17%), with 12% of patients requiring transfusion or erythropoiesis-stimulating agents and any grade hypoxia occurred at 7%. Although largely reversible, these toxicities assume greater significance in a no-evidence-of-disease population. Quality-of-life data will be essential to fully contextualize these findings.

## LITESPARK-011: belzutifan plus lenvatinib after PD-(L)1 failure

Prior to LITESPARK-011, the post-PD-(L)1 treatment landscape was shaped by sequential TKI therapy. CONTACT-03 and TiNivo-2 demonstrated that continuing PD-(L)1 therapy after frontline failure did not improve efficacy and, in some cases, increased toxicity.^5^^,^^6^ Cabozantinib from CONTACT-03 established a benchmark with an ORR of 41% and a median PFS of 10.8 months.^5^ The investigator-initiated LenCabo trial demonstrated that lenvatinib plus everolimus produced an ORR of 52.6% and a median PFS of 15.7 months in a similar population, with a median duration of response (DOR) of approximately 14 to 15 months across both regimens (Table 1).^7^

LITESPARK-011 was designed to test whether combining belzutifan with lenvatinib in an earlier line could mitigate the primary resistance of 34% in LITESPARK-005 while preserving its durability advantage. LITESPARK-011 randomized 747 patients who had progressed on at least one prior PD-(L)1-based regimen to belzutifan 120 mg plus lenvatinib 20 mg daily, or cabozantinib 60 mg daily, with dual primary endpoints of PFS and OS.^8^ Patients could not have had more than two prior regimens. Approximately 70% of patients were enrolled in the second-line setting and 43% had no prior TKI exposure, positioning this largely as a second-line trial.

The study met its primary endpoint of PFS (HR 0.70; 95% CI, 0.59-0.84; *P* < .001), with median PFS of 14.8 lenvatinib and belzutifan versus 10.7 months for cabozantinib. The ORR was 52.6% with lenvatinib and belzutifan versus 40% with cabozantinib, consistent with prior lenvatinib-based combinations and in fact was the same ORR from the recent phase 2 lenvatinib combination with everolimus arm from the LenCabo.^7^

Where this trial was most impactful was in the near doubling of the duration of response from 12 to 23 months with lenvatinib and belzutifan. The OS remains immature with early trends favoring the combination lenvatinib and belzutifan (HR 0.85; 95% CI, 0.68-1.05; *P* = .06) (Table 1). Notably, survival curves, both PFS and OS, overlap for the first 6 months before separating, suggesting that aggressive early disease biology may attenuate the treatment signal initially, before patients with a HIF-2α sensitive biology drive the eventual separation favoring belzutifan plus lenvatinib. Toxicity profiles were comparable between arms, with expected class-specific adverse events from both TKI and HIF-2α inhibition. The combination strategy appears to mitigate the primary resistance observed with belzutifan monotherapy while preserving durability, supporting its use in earlier lines of therapy.

## Conclusion

LITESPARK-011 and LITESPARK-022 collectively advance the case for earlier integration of HIF-2α inhibition in RCC. In the post-PD(L)-1 setting, belzutifan plus lenvatinib improves PFS, ORR, and duration of response compared to cabozantinib, with a comparable rate of adverse events. In the adjuvant setting, the DFS benefit and durable curve separation are compelling, but the doubling of high-grade toxicity in a surgically cured population and the absence of mature OS data require careful interpretation. Both trials reinforce the same core message: HIF-2α targeting delivers real biologic activity and moving it earlier in the disease course may enhance durability. Patient selection will be critical while we await OS maturation.

## Data Availability

Data in the above spotlight piece was taken from GU ASCO 2026 presentations. Table 1 and Graphical Abstract were created with the use of AI.
